# Virtual Screening and Molecular Dynamics Simulation Targeting the ATP Domain of African Swine Fever Virus Type II DNA Topoisomerase

**DOI:** 10.3390/v17050681

**Published:** 2025-05-07

**Authors:** Rui Zhao, Lezi Hou, Weldu Tesfagaber, Linfei Song, Zhenjiang Zhang, Fang Li, Zhigao Bu, Dongming Zhao

**Affiliations:** 1 College of Veterinary Medicine, Xinjiang Agricultural University, Urumqi 830052, China; 19983452130@163.com; 2 State Key Laboratory for Animal Disease Control and Prevention, National African Swine Fever Para-Reference Laboratory, National High Containment Facilities for Animal Diseases Control and Prevention, Harbin Veterinary Research Institute, Chinese Academy of Agricultural Sciences, Harbin 150069, China; welduvet@gmail.com (W.T.); songlf9471@163.com (L.S.); zhangzhenjiang@caas.cn (Z.Z.); lifang201@163.com (F.L.);; 3 College of Veterinary Medicine, Henan Agricultural University, Zhengzhou 450046, China

**Keywords:** ASFV, type II DNA topoisomerase, ATPase domain, small molecule inhibitors, virtual screening

## Abstract

African Swine Fever Virus (ASFV) Topo II ATPase domain, resistant to conventional inhibitors (e.g., ICRF-187) due to M18/W19 steric clashes, was targeted via hierarchical virtual screening (Schrödinger) of the Chembridge library combined with MM/GBSA calculations. Five ligands (10012949, 40242484, 46712145, 15880207, and 33688815) showed high affinity, with 46712145 adopting symmetrical π–π stacking, hydrogen bonds, and alkyl interactions to bypass steric hindrance. Molecular dynamics simulations (100 ns) revealed ligand-induced flexibility, evidenced by elevated RMSD/Rg values versus the free protein. DCCM analysis highlighted enhanced anti-correlated motions between GHKL motifs and sensor domains in chain B/C, suggesting stabilization of a non-catalytic conformation to inhibit ATP hydrolysis. Free energy landscape (FEL) analysis showed 46712145 occupying a broad, shallow energy basin, enabling conformational adaptability, contrasting the narrow deep well of the free protein. This study proposes a symmetric ligand design strategy and conformational capture mechanism to block ATPase activity. Compound 46712145 demonstrates stable binding and dynamic regulation, providing a novel lead scaffold for anti-ASFV drug development. These findings establish a structural framework for combating ASFV through targeted ATPase inhibition.

## 1. Introduction

African Swine Fever (ASF) is an acute and highly lethal infectious disease caused by the African Swine Fever Virus (ASFV), which severely impacts the global pig farming industry [1]. Currently, there are no vaccines or therapeutic drugs available for ASF, necessitating the implementation of biosecurity controls integrated with diagnostic measures [2,3,4]. ASFV is a double-stranded DNA virus, and like eukaryotes and some large nuclear viruses, its genome replication and transcription rely on type II topoisomerase (Topo II) [5]. Topo II unwinds and cleaves DNA strands to alleviate supercoiling, ensuring proper viral genome replication and segregation. Studies reveal that ASFV Topo II initiates expression during early infection 2 h post-infection (hpi) and peaks during active viral DNA synthesis (at 16 hpi). Loss of its function reduces viral transcription by 89%, decreases viral factory formation by 75.5%, and ultimately diminishes viral yield by 99.7% [6].

Recent studies have revealed the cryo-EM structure of ASFV topoisomerase [7,8]. Topo II contains three substructures: the ATPase domain (N-gate), the DNA cleavage core (DNA-gate), and the C-gate domain. These substructures work together through the coordinated opening and closing of the three “gates” (N-gate, DNA-gate, and C-gate) to regulate DNA topology. The N-terminal ATPase domain induces dimerization upon ATP binding, closing the N-gate to capture DNA as the starting point for the reaction. This is followed by DNA unwinding and cleavage, which alleviates DNA supercoiling and ensures the correct replication and distribution of the viral genome. Therefore, Topo II is a key target for antiviral drug development.

In the field of anticancer drug research, small molecule inhibitors targeting the ATPase domain, such as ICRF-187 and ICRF-193, have made significant progress. These inhibitors capture the ATPase domain in a closed conformation, preventing ATP hydrolysis and thus inhibiting the DNA unwinding and relaxation functions of Topo II, ultimately affecting the normal replication and repair of DNA [9,10]. ICRF-187 and ICRF-193 show strong inhibition of Topo II in various eukaryotes and have been used in cancer treatment. However, despite showing significant effects on Topo II of other pathogens, ICRF-187 has limited efficacy against ASFV Topo II. A previous study indicated that the ATPase domain of ASFV Topo II contains unique variable residues (such as MET18 and TRP19), which cause significant steric hindrance between these residues and the piperazine-2,5-dione ring of ICRF-187, preventing effective binding [11]. This finding highlights the need to develop more precise inhibitors specifically targeting ASFV Topo II.

This study employs virtual screening and molecular dynamics simulation techniques to explore potential inhibitors for ASFV Topo II. Using computer-aided drug design (CADD), compounds that may bind to the same site as ICRF-187 on Topo II were screened. These compounds were further validated for stability and inhibitory effects using molecular dynamics simulations. This approach effectively accelerates drug screening and provides theoretical and experimental support for the development of anti-ASFV drugs.

## 2. Results

### 2.1. Three-Dimensional Structure of Topo II ATP Domain and Pocket Analysis

**Figure 1:**
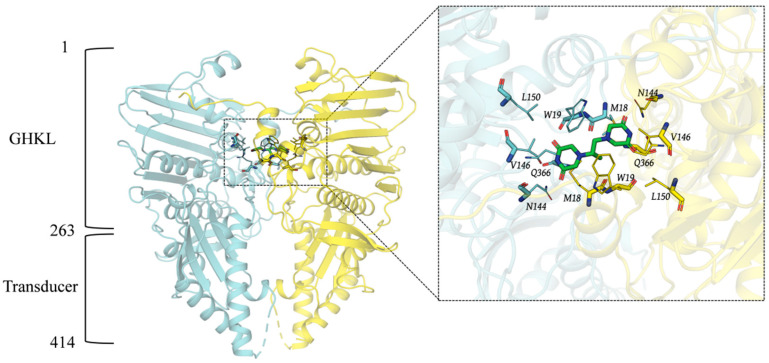
Structure of Topo II ATPase and the location of its binding pocket.

The ASFV Topo II ATP domain consists of an N-terminal GHKL domain (residues 1–263) and a C-terminal transducer domain (residues 264–414), which together form a heart-shaped dimeric complex (Figure 1). Structural comparison indicates that the overall structure of ASFV Topo II ATP domain is highly similar to that of the yeast Topo II ATP domain (RMSD: 1.588 Å), with two non-conserved amino acid residues (MET18 and TRP19) observed at the ICRF-187 binding site. Based on the ICRF-187 binding site in the yeast Topo II ATP domain, we defined the binding pocket of the ASFV Topo II ATP domain for subsequent virtual screening.

### 2.2. Virtual Screening Results from Triple Docking

**Figure 2:**
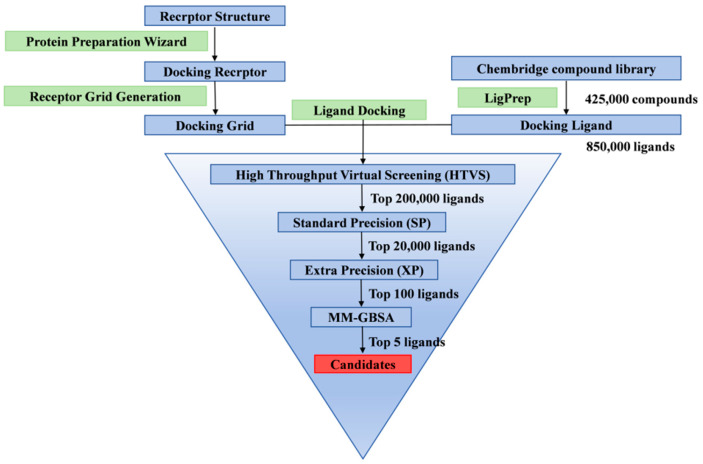
Virtual screening workflow of ASFV Topo II ATPase inhibitors.

After three rounds of screening (HTVS, SP, and XP), the top 100 ranked small molecules were estimated for their binding free energy (ΔG Binding) by MM-GBSA (Figure 2). The results showed that 34 small molecules had ΔG Binding values lower than −50 kcal/mol, with the lowest value being −74.43 ± 5.67 kcal/mol. Based on visual inspection of the binding conformations, three small molecules with strong binding affinities and distinct binding modes were selected as candidate inhibitors for the Topo II ATPase domain. Their XP docking scores and MMGBSA-estimated binding free energies are listed in Table 1.

**Figure 3:**
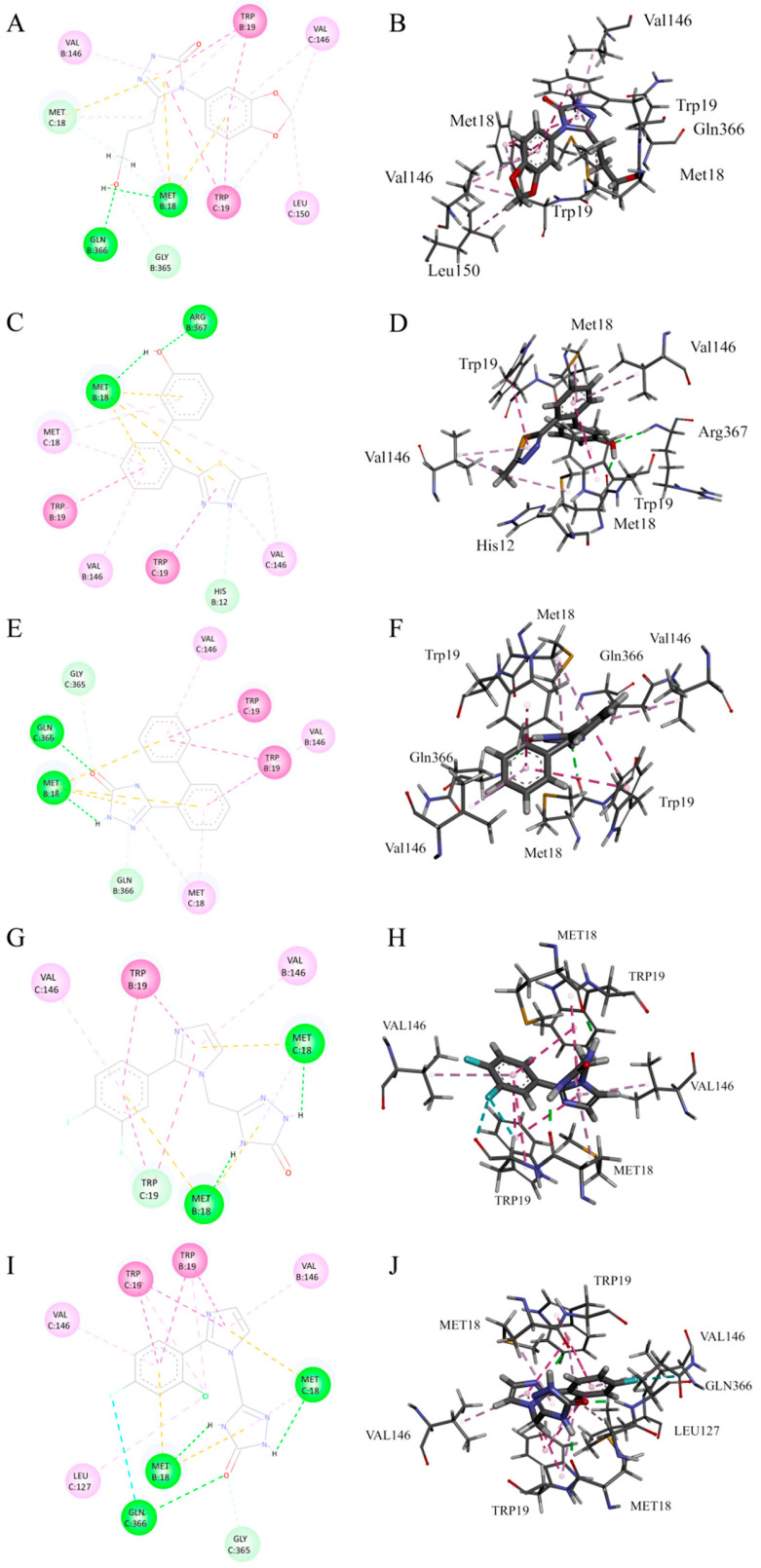
Two-dimensional and 3D interactions of cpd1 (**A**,**B**), cpd2 (**C**,**D**), cpd3 (**E**,**F**), cpd4 (**G**,**H**), and cpd5 (**I**,**J**) with Topo II ATPase.

Molecular docking analysis revealed that cpd1 (Chembridge ID 10012949) predominantly forms strong interactions through classical hydrogen bonds with B chain GLN366 and B chain MET18, while establishing weak interactions with B chain GLY365 and C chain MET18 via carbon–hydrogen bonds (Figure 3A,B). Additionally, alkyl or π-alkyl interactions are observed between C chain LEU150 and the small molecule. Cpd2 (Chembridge ID 40242484) forms strong interactions through classical hydrogen bonds with B chain MET18 and B chain ARG367 within the binding pocket, establishes weak interactions with B chain HIS12 via carbon–hydrogen bonds, and generates alkyl or π-alkyl interactions with C chain MET18 (Figure 3C,D). Cpd3 (Chembridge ID 46712145) forms significant strong interactions through classical hydrogen bonds with B chain MET18 and B chain GLN366 in the binding pocket, while producing weak interactions with B chain GLN366 and C chain GLY365 through carbon–hydrogen bonds (Figure 3E,F). Cpd4 (Chembridge ID 15880207) establishes strong interactions via classical hydrogen bonds with MET18 from both B and C chains in the binding pocket, and forms weak interactions with C chain TRP19 through carbon–hydrogen bonds (Figure 3G,H). Cpd5 (Chembridge ID 33688815) generates strong interactions through classical hydrogen bonds with B chain MET18, C chain MET18 and C chain GLN366, creates weak interactions with C chain GLY365 via carbon–hydrogen bonds, and further forms halogen bonds between the fluorine atom and C chain GLN366 (Figure 3I,J).

Further examination of binding modes demonstrated that the sulfur atoms in MET18 residues from both B and C chains consistently form non-covalent interactions with the π-electron cloud of aromatic rings in all candidate compounds. TRP19 from both B and C chains enhances stable binding of small molecules through π–π stacking or π–π T-shaped interactions, while VAL146 residues from both chains make crucial contributions to complex stability via alkyl or π-alkyl interactions. The symmetry displayed in these interactions indicates that the ASFV Topo II binding pocket exhibits a preference for accommodating small molecule ligands with symmetric structural features.

### 2.3. Molecular Dynamics Simulation Analysis of Candidate Small Molecules with ATP Domain Complexes

**Figure 4:**
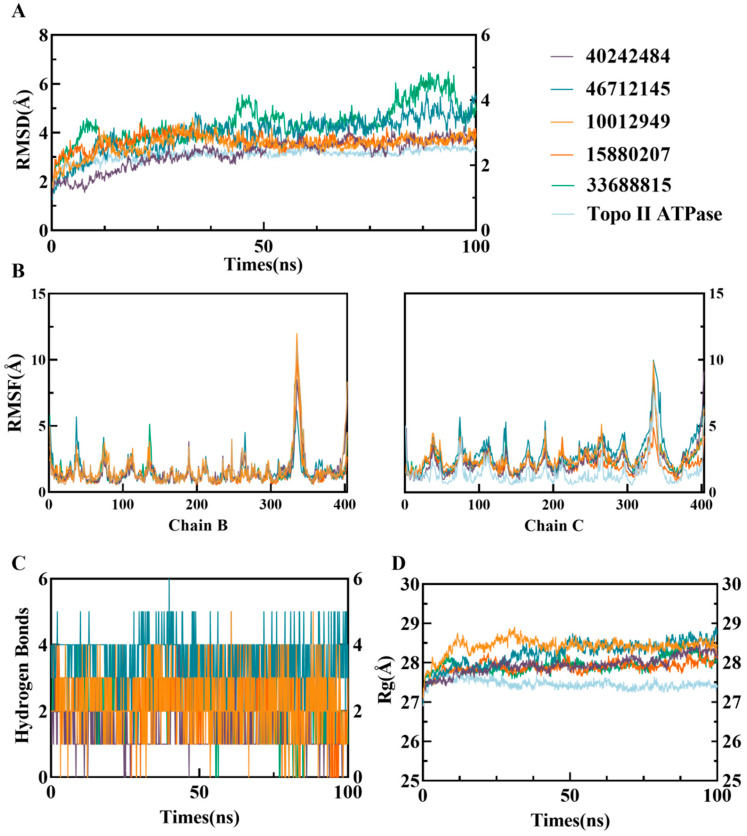
Molecular dynamics simulation analysis of free Topo II ATPase and its complexes with five compounds over 100 ns.

To analyze the dynamic stability of the complexes, the RMSD of the protein backbone atoms was calculated as a function of simulation time (Figure 4A). The RMSD of the backbone atoms of all four systems remained stable after convergence. During the first 35 ns of simulation, the RMSD of the three complexes increased rapidly. Afterward, the RMSD of the cpd1, cpd2, and cpd4 systems fluctuated within a narrow range, with average RMSD values of 3.61 ± 0.04 Å, 3.50 ± 0.08 Å, and 3.63 ± 0.09 Å, respectively. In contrast, the cpd3 and cpd 5 system exhibited larger fluctuations, with the average RMSD of 4.34 ± 0.18 Å and 4.40 ± 0.57 Å, indicating that they may have acquired higher flexibility after equilibration. Furthermore, the RMSD values of the three complexes were all higher than that of the Apo Topo II ATPase (3.21 ± 0.01 Å).

Time evolution analysis of the Rg showed that all small molecule complexes exhibited fluctuations during the first 35 ns of simulation, after which they reached a stable state (Figure 4D). During equilibrium, the Rg values of three complexes remained within a narrow range, with amplitudes below 0.1 Å. The average equilibrium Rg values of these complexes were 28.44 ± 0.01 Å, 28.03 ± 0.03 Å, 28.41 ± 0.03 Å, 27.89 ± 0.02 Å, and 27.95 ± 0.04 Å, respectively, all significantly higher than the Rg value of the apo protein (27.42 ± 0.01 Å).

Molecular dynamic simulations also analyzed the number of hydrogen bonds formed between the ligands and the protein to assess the strength and stability of intermolecular interactions (Figure 4C). The results showed that the cpd3 system formed the most hydrogen bonds throughout the simulation, while the cpd2 system formed the fewest. After equilibration (post 35 ns), the average number of hydrogen bonds formed by three complex systems were 2.18 ± 0.81, 1.30 ± 0.27, 3.4 ± 0.75, 2.66 ± 0.58, and 2.04 ± 0.51, respectively. This suggests that the cpd3 exhibited the most significant hydrogen bond interactions with the target protein, potentially playing an important role in the stability of the system, while the binding of the cpd2 primarily depended on other non-hydrogen bond interactions.

RMSF values calculated over 100 ns of molecular dynamics simulations showed that the flexibility of the amino acid residues of the B chain of Topo II ATPase in all three complexes was consistent with that of the free Topo II ATPase, whereas the flexibility of the C chain was significantly increased (Figure 4B). In particular, the RMSF values of the cpd3 system were higher than the other four, which was consistent with the RMSD results. This suggests that the binding of the small molecules has induced some degree of separation in the double-chain structure of Topo II ATPase.

### 2.4. Free Energy Landscape and Dynamic Cross-Correlation Matrix Analysis

**Figure 5:**
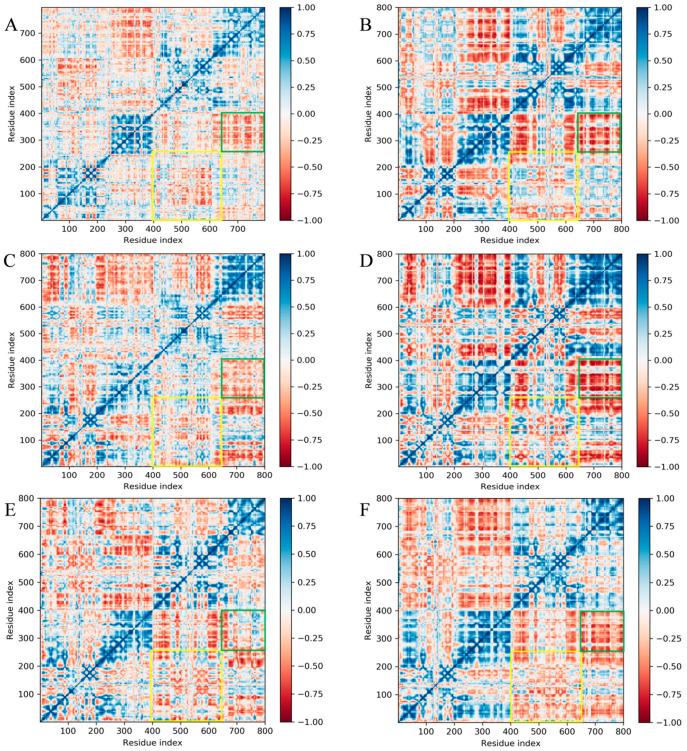
DCCM analysis of Cα residues for Apo Topo II ATPase (**A**) and its complexes with cpd1 (**B**), cpd2 (**C**), cpd3 (**D**), cpd4 (**E**), and cpd5 (**F**). Yellow box and green box represent GHLK domain and Transduce domain.

To specifically examine the relative movement of residues in the B and C chains during the simulation of the Topo II ATPase system, a cross-correlation analysis of Cα atomic fluctuations was performed for all systems, and a Dynamic Cross-Correlation Matrix (DCCM) was plotted (Figure 5). Positive values in the DCCM indicate correlated motion, while negative values indicate anticorrelated motion. The positive and negative correlations in the five compound systems were significantly stronger than those in the free Topo II ATPase system, suggesting that the stability of the complexes in the small molecule systems was enhanced. Further analysis revealed that the GHKL region (yellow box) and transducer region (green box) of the B and C chains in all systems displayed an overall negative correlation, indicating that the B and C chains underwent opposite motions during the simulation.

**Figure 6:**
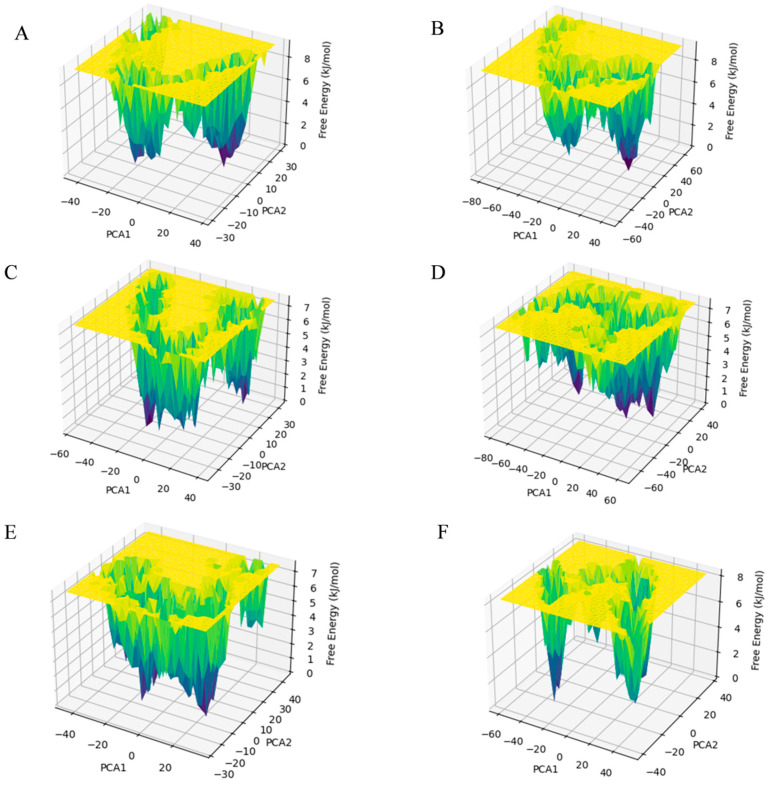
FEL analysis of Apo Topo II ATPase (**A**) and its complexes with cpd1 (**B**), cpd2 (**C**), cpd3 (**D**), cpd4 (**E**), and cpd5 (**F**).

Free energy landscape (FEL) is crucial for understanding the energy stability and conformational states of protein–ligand complexes. Using the first two principal components obtained from PCA, the Gibbs free energy for each conformation of the protein was calculated. Figure 6 shows a contour plot representing the transition from high-energy states to low-energy states, highlighting the energy minima. These minima are represented by a gradient from yellow to green to deep blue, with the deep blue points indicating the most stable states in each system. It can be observed that the energy well formations of the cpd1 and cpd2 systems are similar to the Apo Topo II ATPase system, while the cpd3, cpd4, and cpd5 systems formed a distinct energy well pattern. The free system had narrower, deeper energy wells with lower conformational flexibility, while the small molecule systems exhibited wider, shallower wells, indicating higher conformational flexibility.

**Figure 7:**
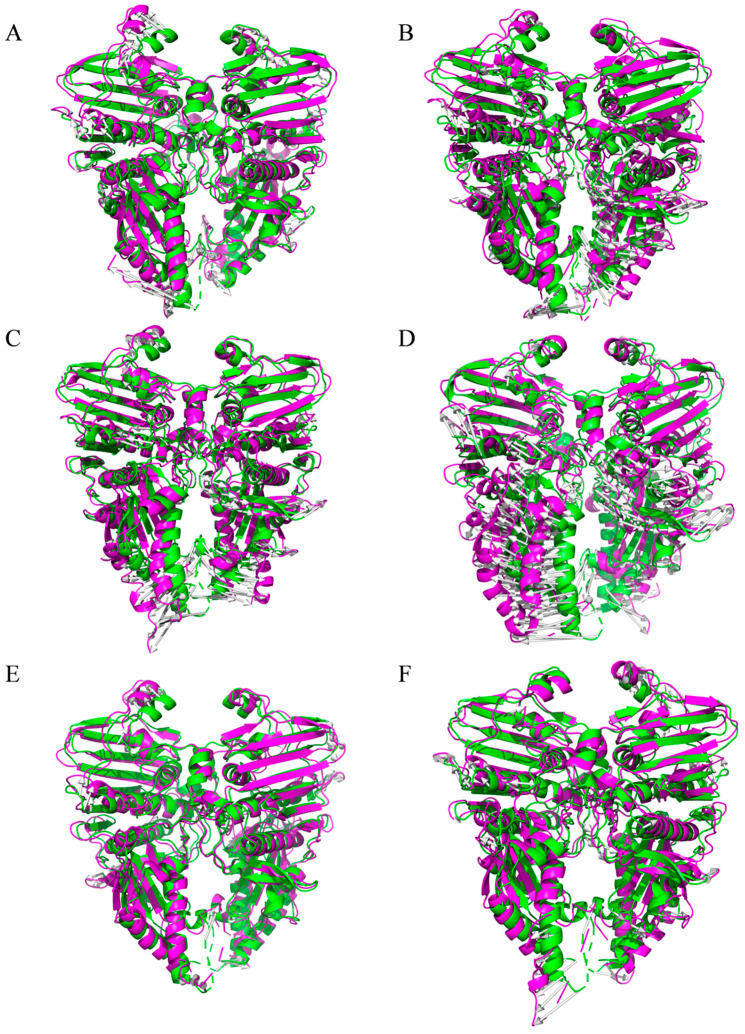
Structural difference analysis of Apo Topo II ATPase (**A**) and complexes with cpd1 (**B**), cpd2 (**C**), cpd3 (**D**), cpd4 (**E**), and cpd5 (**F**).

Subsequently, the lowest energy conformations from the energy wells of each system were extracted from the trajectory (purple) and compared to the initial conformations (green) at the start of the simulation (Figure 7). A structural comparison plot was generated. All systems displayed a trend of reverse separation of the B and C chains, with the free Topo II ATPase system showing the least structural change during the simulation, while the cpd3 system exhibited the greatest structural change, particularly in the transducer region of the B/C chains, consistent with the DCCM results. The RMSD values between the initial and lowest energy conformations for each system were 2.640 Å, 3.288 Å, 2.837 Å, 3.849 Å, 2.502 Å, and 3.151 Å, respectively.

### 2.5. ADME Prediction

The ADME properties of the three compounds obtained through screening were predicted using ADMETlab 3.0. The ADMET properties include molecular weight, topological polar surface area (TPSA), the logarithm of the aqueous solubility value (logS), the logarithm of the n–octanol/water distribution coefficient (logP), the logarithm of the n–octanol/water distribution coefficients at pH = 7.4 (logD), Caco–2 permeability (Caco—2), blood–brain barrier permeability (BBB), and plasma clearance (CLplasma). The ADME results are summarized in Table 2. In addition, all three compounds comply with the Lipinski’s rule.

## 3. Discussion

This study systematically investigated small molecule inhibitors targeting the ATPase domain of ASFV Topo II through virtual screening and molecular dynamic simulations. We pioneered the application of the combined DCCM and FEL analytical strategies for ASFV targets. Five candidate compounds (Chembridge IDs 10012949, 40242484, 46712145, 15880207, and 3368815) were identified with detailed characterization of their binding properties and conformational regulation mechanisms. MM/GBSA calculations revealed binding free energies of −70 ± 5.64, −73.88 ± 5.78, −74.43 ± 5.67, −65.25 ± 7.07, and −63.05 ± 7.08 kcal/mol for these compounds, respectively. Notably, while these values were less favorable than the inhibitor Doxorubizen (−90.71 kcal/mol) calculated using the same methodology in prior study [12], they exhibited stronger binding affinity compared to the positive control doxorubicin (−57.84 kcal/mol) and outperformed previously reported inhibitors 4b (ΔGbind = −41.72 kcal/mol) and 4j (ΔGbind = −40.46 kcal/mol) from independent investigations [13].

Crucially, the identified compounds demonstrated favorable interactions with TRP19 and MET18 residues, which are known to cause steric hindrance in the binding failure of the established inhibitor ICRF-187. Binding mode analysis further supported the hypothesis of symmetric ligand preference within the ASFV Topo II ATPase binding pocket, suggesting that symmetric ligands may enhance binding efficiency through multi-chain synergistic effects. This discovery not only provides new insights into addressing species selectivity challenges in current inhibitors but also offers a strategic framework for developing next-generation antiviral agents targeting ASFV Topo II ATPase.

Molecular dynamics simulation results showed that the candidate small molecules significantly altered the dynamic characteristics of the target protein by inducing conformational separation (e.g., reverse movement of the B/C chains). The increased RMSD and Rg values of the complex systems suggested that the ligands may interfere with the dimeric cooperative mechanism required for ATP hydrolysis. DCCM and FEL analysis indicated that these compounds may lock the ATPase in an inactive conformation by stabilizing the negative correlated motion between the GHKL region and the transducer region, which could inhibit ATP hydrolysis. Among them, cpd3 demonstrates the most optimal binding stability and allosteric effects. This finding is similar to the “conformational capture” strategy of eukaryotic Topo II inhibitors but is the first validation of dynamic regulation feasibility in the ASFV target [10], providing new insights into the study of allosteric inhibition mechanisms.

However, there are certain limitations in this study. The accuracy of force field models (such as OPLS_2005) in describing small-molecule polarity and π interactions may affect binding free energy estimation. Future research could optimize this issue using polarizable force fields (such as AMOEBA) [14]. The simulation duration of 100 ns may be insufficient to capture the slow conformational changes in the target protein, so extending the simulation to 500 ns with enhanced sampling techniques (e.g., Metadynamics) [15] is recommended. Future studies should validate the IC50 values of candidate molecules through in vitro ATPase activity inhibition experiments and clarify binding details through X-ray crystallography.

While this study pioneered the development of dimerization interface-targeting inhibitors for ASFV Topo II ATPase, it did not comprehensively address other druggable sites within this multifunctional enzyme, particularly ATP-binding competitive inhibitors that may exploit conserved catalytic motifs [16]. Future investigations should implement ensemble docking strategies targeting both dimerization and catalytic sites to enable multi-parametric optimization of binding specificity and allosteric effects.

Notably, emerging evidence suggests broader targeting possibilities: Recent crystallographic studies revealed that m-AMSA, a canonical eukaryotic Topo II inhibitor, exhibits unexpected potency against ASFV pP1192R [17]. This dual-action mechanism, involving stabilization of non-covalent Topo II-DNA intermediates, suggests that polypharmacological targeting across multiple functional domains.

## 4. Materials and Methods

### 4.1. Three-Dimensional Structure of ASFV Topo II ATP Domain and Pocket Analysis

First, the Yeast Topo II ATPase-ICRF-187 complex (PDB ID: 1QZR) and ASFV Topo II ATPase (PDB ID: 8WWO) structures were aligned using PyMOL. The small molecule ICRF-187 was retained, and the protein structure of Yeast Topo II ATPase was removed, resulting in the ASFV Topo II ATPase-ICRF-187 complex structure file. Subsequently, the complex structure was processed using the Protein Preparation Wizard module from Schrödinger 2022-3 suite: Hydrogen atoms were added, missing side chains and loops were repaired, crystal water molecules were removed, the protonation state was generated, hydrogen bond networks were optimized, and energy minimization was performed. Finally, the Receptor Grid Generation module was used to define the binding pocket at the position of the small molecule ligand ICRF-187 and to generate the docking box file.

### 4.2. Compound Library Preparation and Generation of Multiple Conformations

The Chembridge compound library (core-part1), provided by Omers Biotech (Shanghai) Co., Ltd., includes 425,000 compounds. Ligand preparation was carried out using the LigPrep module under the following conditions: (1) the OPLS4 force field was applied; (2) all possible ionization states at pH 7.0 ± 2.0 were generated using Ionizer; (3) salt-free ligands were selected; (4) two tautomeric forms were generated for each ligand; and (5) the lowest energy conformation was selected for each ligand. The prepared ligands were then used for virtual screening.

### 4.3. Molecular Docking and MMGBSA-Based Screening of Small Molecule Compounds

Virtual screening was performed using the Glide module with a hierarchical structure-based approach. During the process, compounds were screened at three different levels based on accuracy, High Throughput Virtual Screening (HTVS), Standard Precision (SP), and Extra Precision (XP). The layered screening approach helps filter out potential ligand poses that are capable of binding precisely to the protein’s active site. After completing the three levels of screening, the compounds were ranked by XP scores. The Prime module was used for further analysis, following default settings, including the OPLS 2005 force field and the VSGB 2.0 solvation model. MMGBSA calculations were performed on the top 100 docking results.

### 4.4. Molecular Dynamics Simulations of Docked Complexes

MD simulations were performed using Desmond 6.3 for the ASFV Topo II ATPase apo structure and three small-molecule-bound complexes for 100 ns. The OPLS_2005 force field was applied to describe atomic interactions within the system. Solvent modeling was performed using the TIP3P water model, and sodium and chloride ions were added to achieve a physiological NaCl concentration of 0.15 M. Before simulation, the system underwent energy minimization using steepest descent (5000 steps) and conjugate gradient (10,000 steps) methods.

Next, each system underwent a staged equilibration process. The first phase was NVT equilibration (300 K, 300 ps), where constant particle number (N), volume (V), and temperature (T) were maintained. The system was subjected to 100 ps of Brownian dynamics with temperature set to 300 K, with constraints applied to solute heavy atoms. This was followed by 100 ps of Brownian dynamics with user-defined constraints to further relax the system. The final 12 ps was Langevin dynamics at 300 K, continuing with constraints on the heavy atoms. The second phase was NPT equilibration (1 bar, 24 ps), where constant particle number (N), pressure (P), and temperature (T) were maintained. The system underwent 12 ps of Langevin dynamics under NPT conditions (300 K, 1 bar), retaining constraints on heavy atoms, followed by 12 ps of unrestrained Langevin dynamics (300 K, 1 bar) to ensure thermodynamic equilibrium.

After equilibration, a 100 ns production MD simulation was performed, and several analyses were conducted. Long-range electrostatic interactions were handled using the Particle Mesh Ewald (PME) method, while bonds involving heavy atoms were constrained using Desmond’s custom constraint algorithm. Short-range interactions were calculated using the Verlet cutoff scheme, with a cutoff distance of 14 Å for both van der Waals and electrostatic interactions. After the simulation, we calculated the RMSD, Root Mean Square Fluctuation (RMSF), Rg, and hydrogen bond counts.

### 4.5. Principal Component Analysis, Free Energy Landscape, and Dynamic Cross-Correlation Matrix Analysis

After completing the above steps, the trajectory files (.dtr) from the MD simulations were converted to GROMACS trajectory files (.xtc) using Schrödinger’s dtr2xtc.py script. Principal Component Analysis (PCA) was then performed to analyze the protein dynamics. The GROMACS gmx_covar module was used to identify the principal components and eigenvalues that describe the essential motions, with the first two principal components, PCA1 and PCA2, extracted. The Gibbs free energy was calculated using the gmx_sham module, and the results were visualized using Python 3.12.2 to generate the protein’s FEL. Additionally, based on the lowest Gibbs free energy frame recorded in the log file, the PDB structure was extracted from Schrödinger, and a visualization of protein motions was generated using PyMOL’s ModeVectors.py script, resulting in a porcupine plot.

For DCCM analysis, the xtc trajectory files were first converted to the NAMD format (.dcd) using the MDTraj library in Python. DCCM analysis was then performed and visualized using the Bio3D R package.

### 4.6. In Silico ADME Prediction

After completing the docking analysis and molecular dynamics (MD) simulation of the hit compounds, we became interested in the ADME properties of these compounds. In order to explore whether they have drug-likeness, we used ADMETlab 3.0 (https://admetlab3.scbdd.com/, accessed on 15 April 2025) [18] to predict their basic physicochemical properties and ADME characteristics.

## Figures and Tables

**Table 1:** Docking scores and binding free energy of the three small molecules.

Chembridge ID	XP GScore	ΔG Binding (kcal/mol)	ΔEvdW (kcal/mol)	ΔEelec (kcal/mol)	ΔGpol (kcal/mol)
10012949	−10.552	−70 ± 5.64	−43.55 ± 2.76	−14.12 ± 4.49	20.79 ± 2.94
40242484	−9.511	−73.88 ± 5.78	−43.39 ± 2.72	−12.37 ± 3.73	22.78 ± 2.27
46712145	−11.929	−74.43 ± 5.67	−42.25 ± 2.42	−18.77 ± 3.8	25.02 ± 2.52
15880207	−9.394	−65.25 ± 7.07	−43.28 ± 3.24	−19.67 ± 4.59	27.71 ± 2.42
33688815	−10.222	−63.05 ± 7.08	−42.6 ± 3.12	−16.41 ± 6.3	25.38 ± 2.48

**Table 2:** ADME analysis of five compounds.

Chembridge ID	MW	TPSA	logS	logD	logP	caco2	BBB	cl-plasma
10012949	263.09	89.37	−2.06	0.69	0.26	−5.17	0.94	4.12
40242484	268.07	46.01	−3.57	3.03	2.84	−4.88	0.65	4.94
46712145	237.09	61.54	−3.14	2.21	2.31	−5.15	0.21	6.61
15880207	277.08	79.36	−3.00	1.27	1.06	−5.52	0.56	5.72
33688815	295.06	70.98	−3.52	1.94	1.53	−5.64	0.07	4.52

TPSA: Compounds in the range from 0 to 140 will be considered proper. LogS: compounds in the range from −4 to 0.5 log mol/L will be considered proper. LogD: compounds in the range from 1 to 3 log mol/L will be considered proper. LogP: compounds in the range from 0 to 3 log mol/L will be considered proper. caco2: compounds with a value greater than −5.15 will be considered excellent. BBB: the compounds are excellent in the range of 0–0.3, moderate in the range of 0.3–0.7, and poor in the range of 0.7–1. cl-plasma: the compounds are excellent in the range of 0–5, moderate in the range of 5–15, and poor when exceeding 15.

## Data Availability

The data that support the findings of this study are available from the corresponding author upon reasonable request.
